# Genome-Wide miRNA Analysis Identifies Potential Biomarkers in Distinguishing Tuberculous and Viral Meningitis

**DOI:** 10.3389/fcimb.2019.00323

**Published:** 2019-09-10

**Authors:** Liping Pan, Fei Liu, Jinli Zhang, Jing Li, Hongyan Jia, Mailing Huang, Xuehua Liu, Weibi Chen, Zeyu Ding, Yajie Wang, Boping Du, Rongrong Wei, Qi Sun, Aiying Xing, Zongde Zhang

**Affiliations:** ^1^Beijing Key Laboratory for Drug Resistant Tuberculosis Research, Beijing Tuberculosis and Thoracic Tumor Research Institute, Beijing Chest Hospital, Capital Medical University, Beijing, China; ^2^Tuberculosis Department, Beijing Tuberculosis and Thoracic Tumor Research Institute, Beijing Chest Hospital, Capital Medical University, Beijing, China; ^3^Neurology Department, Chinese People's Liberation Army 263 Hospital, Beijing, China; ^4^Hyperbaric Oxygen Department, Beijing Chao-Yang Hospital, Capital Medical University, Beijing, China; ^5^Neurology Department, Xuanwu Hospital, Capital Medical University, Beijing, China; ^6^Neurology Department, Beijing Tiantan Hospital, Capital Medical University, Beijing, China; ^7^Laboratory Medical Center, Beijing Ditan Hospital, Capital Medical University, Beijing, China

**Keywords:** tuberculous meningitis, viral meningitis, diagnosis, genome-wide microarray, miRNA

## Abstract

Tuberculous meningitis (TBM) is the most common and severe form of central nervous system tuberculosis. Due to the non-specific clinical presentation and lack of efficient diagnosis methods, it is difficult to discriminate TBM from other frequent types of meningitis, especially viral meningitis (VM). In order to identify the potential biomarkers for discriminating TBM and VM and to reveal the different pathophysiological processes between TBM and VM, a genome-wide miRNA screening of PBMCs from TBM, VM, and healthy controls (HCs) using microarray assay was performed (12 samples). Twenty-eight differentially expressed miRNAs were identified between TBM and VM, and 11 differentially expressed miRNAs were identified between TBM and HCs. The 6 overlapping miRNAs detected in both TBM vs. VM and TBM vs. HCs were verified by qPCR analysis and showed a 100% consistent expression patterns with that in microarray test. Statistically significant differences of 4 miRNAs (miR-126-3p, miR-130a-3p, miR-151a-3p, and miR-199a-5p) were further confirmed in TBM compared with VM and HCs in independent PBMCs sample set (*n* = 96, *P* < 0.01). Three of which were also showed significantly different between TBM and VM in CSF samples (*n* = 70, *P* < 0.05). The receiver operating characteristic curve (ROC) analysis showed that the area under the ROC curve (AUC) of these 4 miRNAs in PBMCs were more than 0.70 in discriminating TBM from VM. Combination of these 4 miRNAs could achieve better discriminative capacity [AUC = 0.893 (0.788–0.957)], with a sensitivity of 90.6% (75.0–98.0%), and a specificity of 86.7% (69.3–96.2%). Additional validation was performed to evaluate the diagnostic panel in another independent sample set (*n* = 49), which yielded a sensitivity of 81.8% (9/11), and specificity of 90.0% (9/10) in distinguishing TBM and VM, and a sensitivity of 81.8% (9/11), and a specificity of 84.6% (11/13) in discriminating TBM from other non-TBM patients. This study uncovered the miRNA profiles of TBM and VM patients, which can facilitate better understanding of the pathogenesis involved in these two diseases and identified 4 novel miRNAs in distinguishing TBM and VM.

## Introduction

Tuberculous meningitis (TBM) is one of the serious chronic infections of central nervous system (CNS) caused by *Mycobacterium tuberculosis* (*M.TB*). About more than half of TBM patients die despite anti-tuberculosis chemotherapy in developing countries (Bidstrup et al., 2002). Delayed diagnosis and treatment are considered as the leading cause of the high mortality and disability in many recent reports (Phypers et al., 2006; Dinnes et al., 2007; Be et al., 2009). Due to the non-specific clinical presentation and lack of efficient diagnosis methods, the diagnosis of TBM remains difficult until now. TBM was definitely diagnosed by Ziehl-Neelsen (ZN) staining of cerebrospinal fluid (CSF) smears and CSF *M.TB* culture. However, the smear has very low sensitivity (10–20%), whilst *M.TB* culture lacks sensitivity and requires appropriately 6–8 weeks obtaining the result (Thwaites et al., 2002; Pai et al., 2003). Although WHO has recommended XpertMTB/RIF test prior to microscopy and culture as the preferred diagnostic test for suspected TBM patients, the use of this test was limited due to the high cost and low positive predictive value in areas with low prevalence of rifampicin resistance (Torok, 2015). The interferon-gamma release assays, the radiological findings (computed tomography or magnetic resonance images), and other CSF laboratory parameters, including adenosine deaminase (ADA), lymphocyte count, glucose, and chloride concentration, are frequently non-specific (Golden and Vikram, 2005; Quan et al., 2006; McNerney et al., 2012). Therefore, identification of novel biomarkers is urgently needed for TBM diagnosis.

Given that the clinical manifestations of TBM are similar with other chronic meningitis, such as viral meningitis, bacterial meningitis, and fungal meningitis (mainly cryptococcal meningitis), the differentiation between TBM and the other chronic meningitis is clinically important. Fortunately, the incidence of bacterial meningitis has been declining in recent years due to the use of antibiotics, and the excellent tests for cryptococcal meningitis have been developed. Thus, the differentiation between TBM and these two diseases were generally uncomplicated. The incidence of viral meningitis (VM) has been estimated between 0.26 and 17 cases per 100,000 dependent on the age or vaccination status of the population (McGill et al., 2017). Despite molecular methods in VM diagnosis have been developed, many cases still remain without a confirmed viral etiology. Thus, the differentiation between TBM and VM was still difficult. Novel diagnostic parameters, which can distinguish these two types of meningitis, are urgently needed.

microRNA (miRNA) is a type of conserved non-coding small RNA at length of 18–25 nucleotides, which regulates gene expression and involved in various biological signaling pathways, including cell proliferation, differentiation, apoptosis, immune response, and angiogenesis. The abnormal expression of miRNA in tissue or blood is closely related to many diseases, including tuberculosis (TB) (Sabir et al., 2018). Until now, the information on genome-wide screening of miRNA in TBM was limited. Only one study has identified differential miRNA profile between TBM and healthy controls (HCs) using microarray (Hu et al., 2019). Another study has validated the association between miR-29 and TBM, and found miR-29 was significantly up-regulated in peripheral blood mononuclear cells (PBMCs), and CSF of pediatric TBM patients than that of healthy controls (Pan et al., 2017). However, there is no study on identifying the differential miRNA profile between TBM and VM patients. The miRNA in PBMCs were stable and high-quality, which can be obtained using conventional RNA extraction methods, and used as diagnostic markers in clinical practice (Sabir et al., 2018). Therefore, in order to identify the potential biomarkers for discriminating TBM and VM, and to reveal the different pathophysiological processes between TBM and VM, a genome-wide miRNA screening of PBMCs from TBM, VM, and HCs was performed and the differentially expressed miRNA were validated in an independent sample set, including PBMCs and CSF.

## Materials and Methods

### Study Population

The study was performed in accordance with the guidelines of the Helsinki Declaration and its later amendments or comparable ethical standards, and was approved by the Ethics Committee of the Beijing Chest Hospital, Capital Medical University.

Suspected TBM and VM patients were enrolled between Sep 2012 and Aug 2019, from Beijing Chest Hospital, Beijing Chao-yang Hospital, Beijing Tiantan Hospital, Beijing Ditan Hospital, Xuanwu Hospital and People's Liberation Army 263 hospital. All recruited patients, or a direct relative for those with an abnormal mental state, gave informed consent to participate in the study. Healthy controls (HCs) were recruited from a TB screening campaign in Beijing between November 2012 and December 2014, and from the volunteers in Beijing Chest Hospital between June 2019 and Aug 2019. Other non-TBM patients were recruited between June 2019 and Aug 2019, from Beijing Chao-yang Hospital, Beijing Tiantan Hospital, Xuanwu Hospital and People's Liberation Army 263 hospital. The TBM, VM, HCs and other non-TBM were defined as previous studies (Li et al., 2017; Sun et al., 2018): (1) TBM: acid-fast bacilli were detected in the CSF, CSF culture was positive, or the CSF XpertMTB/RIF test was positive for *M.TB*. (2) VM: a viral etiology was confirmed, or the clinical outcome was favorable with supportive and/or antiviral therapy, and bacterial, fungal and other non-infectious causes of meningitis (malignancy, neurosarcoidosis, autoimmune disorders) were ruled out (Hristea et al., 2012; Li et al., 2017). (3) HCs: persons without any clinical symptoms of diseases, and showed negative TST and T-SPOT. *TB* tests, normal chest radiograph. (4) Other non-TBM: an alternative diagnosis was made, without convincing signs of combining TBM or VM. Individuals with positive hepatitis B virus (HBV) or hepatitis C virus (HCV), positive human immunodeficiency virus (HIV), malignancies, severe autoimmune diseases, diabetes, or those who took immunosuppressive or immunopotentiator agents, or were in pregnancy, or lactation were excluded.

### Specimen Collection

Peripheral blood samples (6 mL) were collected in heparin-containing vacutainer tubes from each subject. PBMCs were separated by density gradient using Lympholyte Cell Separation Media (Tianjin Haoyang Biological Manufacture Co., Ltd, China) within 6 h of blood collection, and then were lysed by QIAZOL reagent immediately, which provided in the miRNeasy® Mini kit (QIAGEN, Germany), and stored at −80°C until use. All CSF samples (3 mL) were collected from patients with suspected tuberculous and viral meningitis during a routine diagnostic workup. After centrifugation at 2000 × g for 10 min at 4°C, the CSF supernatant was further centrifuged at 16,000 × g for 10 min at 4°C, and aliquoted into sterile polypropylene microtubes and stored at −80°C until use.

### RNA Extraction

Total RNA was extracted from PBMCs using the miRNeasy® Mini kit (QIAGEN, Germany) according to the protocols recommended by the manufacturer. Total RNA was extracted from CSF (200 μL) using the miRNeasy Serum/Plasma kit (QIAGEN, Germany) according to the protocols recommended by the manufacturer, and the exogenous non-human spike-in cel-miR-39-3p (5.6 × 10^8^ copies) was added simultaneously (QIAGEN, Germany). RNase-free DNase I (QIAGEN, Germany) was added to remove the genomic or cell-free DNA contamination. The integrity and quality of RNA from PBMCs was evaluated using an Agilent 2100 Bioanalyzer (Agilent Technology). RNA with a 2100 RIN (RNA integrity number) ≥ 7.0 and 28S/18S > 0.7 was used for the microarray study and qPCR validation. The quality of RNA from CSF was evaluated by spectrophotometry according to the absorbance (A) at 260 and 280 nm, respectively. Only CSF miRNA samples with A260/A280 ratios between 1.80 and 2.10 were used for qPCR validation.

### Microarray Test and Bioinformatics Analysis

miRNA molecular in total RNA was labeled by miRNA Complete Labeling and Hyb Kit (Agilent technologies, Santa Clara, CA, USA) followed the manufacturer's instructions. Each slide (8^*^60 K Agilent Human miRNA Microarray, version 19.0) was hybridized with 100 ng Cy3-labeled RNA using miRNA Complete Labeling and Hyb Kit (Agilent technologies, Santa Clara, CA, USA) in hybridization Oven (Agilent technologies, Santa Clara, CA, USA) at 55°C, 20 rpm for 20 h according to the manufacturer's instructions, hybridization section. After hybridization, slides were washed in staining dishes (Thermo Shandon, Waltham, MA, USA) with Gene Expression Wash Buffer Kit (Agilent technologies, Santa Clara, CA, USA). Slides were scanned by Agilent Microarray Scanner (Agilent technologies, Santa Clara, CA, USA), and Feature Extraction software 10.7 (Agilent technologies, Santa Clara, CA, USA) with default settings. Raw data were normalized by Quantile algorithm, Gene Spring Software 11.0 (Agilent technologies, Santa Clara, CA, USA). The microarray experiments were performed by following the protocol of Agilent technologies Inc. at Shanghai Biotechnology Corporation. The microarray data was deposited in GEO database: GSE131708.

Fold changes of miRNA expression values were calculated between TBM and the other two groups. Differentially expressed miRNAs (*P*-value <0.05 and with a fold change of at least 1.5 or more) were identified and chose for further analysis.

### Quantitative Real-Time PCR Analysis

For miRNA analysis in PBMCs, a total of 100 ng of purified RNA was reverse transcribed to cDNA using TaqMan™ MicroRNA Reverse Transcription kit (Applied Biosystems, Inc., USA) and the stem-loop primers in TaqMan® MicroRNA Assays (Applied Biosystems, Inc., USA) according to the manufacturer's protocol. Target cDNAs were then mixed with TaqMan® Universal PCR Master Mix and probes in TaqMan® MicroRNA Assays (Applied Biosystems, Inc., USA). Quantitative real-time PCR was performed on the ABI 7900 Real-time PCR System (Applied Biosystems, Inc. USA). Expression threshold for each miRNA detector was automatically determined.

For miRNA analysis in CSF, multiplex RT, and pre-amplification were performed in order to increases the target abundance without introducing bias (Murray et al., 2011). Briefly, an equal volume of all stem-loop primers of target miRNAs in TaqMan® MicroRNA Assays (Applied Biosystems, Inc., USA) were mixed and diluted to 0.05 × with 1 × Tris-EDTA buffer (pH 8.0), and then a total of 100 ng of purified RNA, 6 μL stem-loop primers mix and other reagents in TaqMan™ MicroRNA Reverse Transcription kit (Applied Biosystems, Inc., USA) were mixed as a final volume of 15 μL for each reaction underwent multiplexed RT, according to the manufacturer's protocol. Next, pre-amplification of abovementioned specific RT products was performed. An equal mix of all 20 × TaqMan assay probes of target miRNAs in TaqMan® MicroRNA Assays (Applied Biosystems, Inc., USA) were mixed and diluted to 0.2 × with 1 × Tris-EDTA buffer (pH 8.0). Pre-amplification reactions (each 25 mL) for each sample contained 12.5 μL of 2 × pre-amplification master mix (Applied Biosystems, Inc., USA), 3.75 μL of the diluted TaqMan assay probe mix, 2.5 μL of multiplexed RT product, and 6.25 μL nuclease-free ddH_2_O. Pre-amplification was performed on the Quantistudio7 Real-time PCR System (Applied Biosystems, Inc. USA) with the follow procedure: After heating to 95°C for 10 min, hold on 55°C for 2 min and 72°C for 2 min, and then 14 cycles of 95°C for 15 s and 60°C for 4 min, afterward 99.9°C for 10 min. The pre-amplification products were diluted 1:10 with nuclease-free ddH_2_O. One μL diluted pre-amplification product was then mixed with 10 μL TaqMan® Universal PCR Master Mix, 1 μL TaqMan probes in TaqMan® MicroRNA Assays (Applied Biosystems, Inc., USA), and 8 μL nuclease-free ddH_2_O to make a final volume of 20 μL quantitative real-time PCR reaction according to the manufacturer's custom protocol. The quantitative real-time PCR reaction was performed on the Quantistudio7 Real-time PCR System (Applied Biosystems, Inc. USA). Expression threshold for each miRNA detector was automatically determined.

We calculated 2^−ΔΔ*CT*^and used this statistic to determine relative gene expression. The relative amount of miRNAs in PBMCs was normalized against U6 snRNA (Applied Biosystems, Inc. USA), and the relative amount of miRNAs in CSF was normalized against a spike-in control cel-miR-39-3p (QIAGEN, Germany) or U6 snRNA (Applied Biosystems, Inc. USA) (Murray et al., 2016).

### Data Analysis

The differentially expressed miRNAs between TBM and the other two groups were analyzed using the *t*-test. The receiver operating characteristic curve (ROC) analysis was conducted to evaluate the discriminative ability of miRNA in discriminating TBM from VM, HCs, and other non-TBM groups, with the overall accuracy assessed by the area under the ROC curve (AUC) values. Logistic regression with forward stepwise analysis was used to further establish the diagnostic panel. Significance was defined as *P* < 0.05. All statistical analysis was performed using the commercial statistical software SPSS version 21.0 (SPSS, Inc., Chicago, IL, USA).

## Results

### Characteristics of the Study Population

A total of 257 suspected TBM and VM patients were recruited. Forty-seven TBM and 44 VM patients satisfied the inclusion and exclusion criteria, were included in the study. In addition, a total of 53 healthy controls were also enrolled. Four samples in each group were selected for microarray analysis. Another 32 TBM, 30 VM, and 34 HCs were assigned to the first validation set. The remaining 11 TBM, 10 VM, and 15 HCs were assigned to the second validation set. Furthermore, 11 non-TBM patients, including 2 bacterium meningitis, 1 brucella meningitis, 4 central nervous system tumors, 4 stroke patients, 1 pulmonary encephalopathy, and 1 epilepsy were also recruited in the second validation set. Demographic information on the study population is summarized in Table 1. There were no significant differences in age or gender between TBM and the other groups (*P* > 0.05).

**Table 1:** Demographic characteristics of the study participants.

**Study complex**	**Characters**	**TBM**	**VM**	**HCs**	**Other non-TBM**	*P*-value^*^	***P*-value^#^**	***P*-value^‡^**
Microarray set	*n*	4	4	4				
	Male/female	2/2	3/1	3/1	–	0.465	0.465	-
	Age (median, range)	40 (18–48)	38 (22–68)	34 (31–39)	–	0.985	0.784	-
First validation set	*n*	32	30	34				
	Male/female	16/16	19/11	13/21	-	0.290	0.336	-
	Age (median, range)	27 (18–70)	29 (18–71)	30 (23–54)	-	0.560	0.943	-
Second validation set	*n*	10	11	15	13			
	Male/female	6/4	7/4	9/6	6/7	0.864	1.000	0.510
	Age (median, range)	24 (17–65)	25 (18–80)	26 (21–32)	37 (18–64)	0.621	0.678	0.054

*TBM, tuberculous meningitis; VM, viral meningitis; HCs, healthy controls; Other non-TBM, including 2 bacterium meningitis, 1 brucella meningitis, 4 central nervous system tumors, 4 stroke patients, 1 pulmonary encephalopathy and 1 epilepsy; n, number of subjects*.

* *TBM vs. VM*.

# *TBM vs. HCs*.

‡ *TBM vs. Other non-TBM*.

### miRNA Microarray Profiling of PBMCs in TBM, VM, and HCs

**Figure 1:**
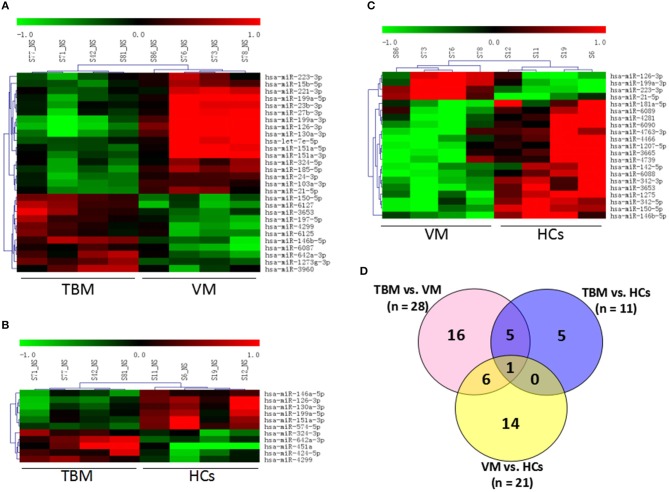
Genome-wide miRNA profile of PBMCs from TBM, VM, and HCs. Unsupervised cluster analysis of differentially expressed miRNAs in **(A)** TBM vs. VM and **(B)** TBM vs. HCs; **(C)** VM vs. HCs; **(D)** Differentially expressed genes with *P* < 0.05 and fold change ≥1.5 in pair-wise comparisons. TBM, tuberculous meningitis; VM, viral meningitis; HCs, healthy controls.

Genome-wide microarray analysis was employed to uncover the miRNA expression profiles of PBMCs from TBM, VM, and HCs. Differential expression was tested by the pair-wise comparison: (i) TBM vs. VM, (ii) TBM vs. HCs, and (iii) VM vs. HCs. The miRNAs with significant changes in expression level in the pair-wise comparison (fold change ≥ 1.5 and *P*-values <0.05) were presented in Figure 1. A total of 28 differentially expressed miRNAs were identified between the TBM and VM group, of which 17 were down-regulated and 11 were up-regulated. Meanwhile, there were 11 differentially expressed miRNAs between the TBM and HCs group, with 6 of which were down-regulated and 5 were up-regulated. Twenty-one differentially expressed miRNAs were identified between the VM and HCs group, of which 17 were down-regulated and 4 were up-regulated. In comparison of TB with the other 2 groups, it shared 6 overlapping miRNAs (miR-126-3p, miR-130a-3p, miR-151a-3p, miR-199a-5p, miR-642a-3p, and miR-4299) with significantly different expression level (Supplementary Table 1). Unsupervised cluster analysis indicated that the differentially expressed miRNAs generated by the pair-wise comparisons could reflect the different statuses of diseases (Figure 1).

### Independent Validation of Differentially Expressed miRNA in PBMCs

In order to validate the microarray results, the 6 overlapping miRNAs with significantly different expression level in both TBM vs. VM and TBM vs. HCs were selected for qPCR analysis, which were performed in the same samples in the microarray test. The fold change values between TBM and other two groups were presented in Table 2. Although the fold change values on qPCR test varied against values obtained from the microarray test, the regulation patterns of these 6 miRNAs in qPCR validation tests showed 100% consistent with the microarray results (Table 2). This difference on fold change values may be due to the different technical principle and different normalization method in data analysis between microarray and qPCR.

**Table 2:** Validation of the 6 overlapping miRNAs between TBM and the other two groups using the same samples in microarray set.

**miRNA**	**Microarray**				**qPCR**			
	**Fold Change (TBM/VM)**	***P*-value**	**Fold Change (TBM/HCs)**	***P*-value**	**Fold Change (TBM/VM)**	***P*-value**	**Fold Change (TBM/HCs)**	***P*-value**
hsa-miR-126-3p	0.220	0.003	0.457	0.014	0.243	0.093	0.448	0.110
hsa-miR-130a-3p	0.244	0.006	0.518	0.041	0.094	0.012	0.227	0.099
hsa-miR-151a-3p	0.418	0.033	0.589	0.047	0.152	0.022	0.254	0.087
hsa-miR-199a-5p	0.386	0.015	0.624	0.042	0.178	0.092	0.410	0.065
hsa-miR-642a-3p	1.656	0.018	1.842	0.008	1.481	0.200	1.106	0.758
hsa-miR-4299	1.611	0.026	1.554	0.014	1.202	0.702	1.664	0.277

*TBM, tuberculous meningitis, n = 4; VM, viral meningitis, n = 4; HCs, healthy controls, n = 4*.

**Figure 2:**
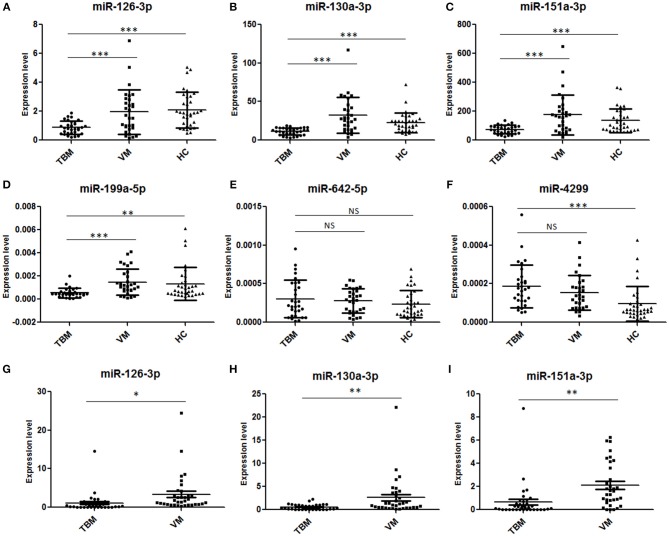
Validation of the 6 overlapping miRNAs in PBMCs and CSF by qPCR in the first independent sample set. TBM, tuberculous meningitis; VM, viral meningitis; HCs, healthy controls. ^*^P < 0.05; ^**^P < 0.01; ^***^P < 0.001; Horizontal bar, the relative expression value of validated miRNAs. The expression values of the validated miRNAs were normalized to the housekeeping miRNA U6 in PBMCs **(A–F)** (TBM, *n* = 32; VM, *n* = 30; HCs, *n* = 34) and spike-in control miRNA cel-miR-39-3p in CSF **(G–I)** (TBM, *n* = 36; VM, *n* = 34; HCs, *n* = 38).

Next, we further validated these 6 miRNAs in an additional independent sample set, which included 32 TBM patients, 30 VM patients, and 34 HCs (Supplementary Table 2 and Figures 2A–F). miRNAs in PBMCs were firstly validated, and the results showed that 4 miRNAs (miR-126-3p, miR-130a-3p, miR-151a-3p, and miR-199a-5p) presented significantly different expression level between the TBM group and the other 2 groups (*P* < 0.01) and the expression patterns were consistent with the microarray study. The miR-4299 also presented significantly different expression level between the TBM group and the HCs group, but no significant difference was detected between the TBM group and VM group; while no significant difference was detected in the expression levels of miR-642-3p in both TBM vs. VM and TBM vs. HCs comparisons.

### Independent Validation of Differentially Expressed miRNA in CSF

To further confirm the results obtained from PBMCs miRNA analysis, the expression levels of miR-126-3p, miR-130a-3p, miR-151a-3p, and miR-199a-5p were measured in CSF samples from total 36 TBM patients and 34 VM patients (Supplementary Table 2 and Figures 2G–I). U6 snRNA and the spike-in control cel-miR-39-3p were used as the housekeeping genes. However, as shown in Supplementary Figure 1, although the amount of total RNA used for reverse transcription were consistent across the samples (100 ng), we found that the expression levels of U6 snRNA in all the 70 CSF samples were not stable and it showed a larger standard deviation (3.30) of cycle threshold (Ct) value across the CSF samples. These results indicated that U6 SnRNA was not appropriate as a housekeeping miRNA for CSF normalization. In contrast, the expression levels of spike-in control cel-miR-39-3p were stable among all the CSF samples and it showed a lower standard deviation (0.70) of Ct value across the CSF samples. Therefore, the relative amounts of the 4 differential miRNAs in CSF were normalized against the spike-in control cel-miR-39-3p. As shown in Supplementary Table 2 and Figure 2, miR-126-3p, miR-130a-3p, and miR-151a-3p all presented significantly lower expression level in the TBM group than that in the VM group (*P* < 0.05), and the regulation patterns were consistent with the results in microarray analysis and further PBMCs validation. No amplification was detected in miR-199a-5p, although the pre-amplification was performed to increase the target abundance.

### Diagnostic Values of miRNAs in Distinguishing TBM From VM and HCs

**Figure 3:**
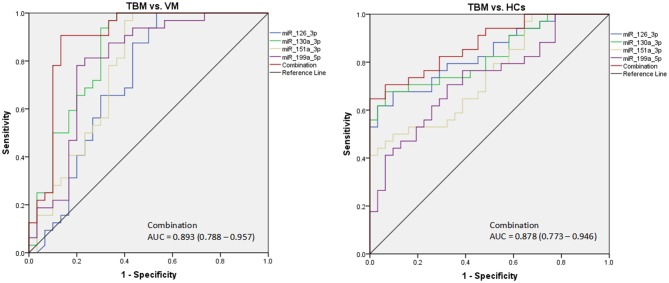
The diagnostic values of the 4 differentially expressed miRNAs in PBMCs in discriminating TBM from VM and HCs. TBM, tuberculous meningitis; VM, viral meningitis; HCs, healthy controls. The receiver operating characteristic curve (ROC) depicts the sensitivity and specificity of the miRNAs in PBMCs (TBM, *n* = 32; VM, *n* = 30; HCs, *n* = 34) in discriminating TBM from VM and HCs.

Due to the significantly different expression of the 4 miRNAs (miR-126-3p, miR-130a-3p, miR-151a-3p, and miR-199a-5p) in PBMCs between TBM and the other 2 groups, we try to evaluate whether these 4 miRNAs or a combination could discriminate TBM from VM and HCs. The receiver operating characteristic curve (ROC) analysis was conducted to assess the discriminative potential of these 4 miRNAs in the PBMCs validation set (Table 3 and Figure 3). The areas under the curve (AUCs) of these 4 miRNAs (miR-126-3p, miR-130a-3p, miR-151a-3p, and miR-199a-5p) were 0.716 (0.587–0.823), 0.836 (0.721–0.918), 0.762 (0.637–0.861), and 0.794 (0.672–0.886), respectively, in discriminating TBM from the VM group. miR-130a-3p was the best miRNA for distinguishing the TBM group from the VM group. The AUC values of these 4 miRNAs (miR-126-3p, miR-130a-3p, miR-151a-3p and miR-199a-5p) were 0.836 (0.723–0.916), 0.827 (0.713–0.910), 0.746 (0.622–0.846), and 0.730 (0.605–0.832), respectively, in discriminating TBM from the HCs group. miR-126-3p presented the best capacity in distinguishing the TBM group from the HCs group. Logistic regression with forward stepwise analysis indicated that the combination of these 4 miRNA could display a better discriminative ability in discriminating TBM from VM group [AUC = 0.893 (0.788–0.957)], and HCs group [AUC = 0.878 (0.773–0.946)]. It exhibited a sensitivity of 90.6% and a specificity of 86.7% in discriminating TBM from the VM group, and a sensitivity of 93.5% and a specificity of 70.6% in discriminating TBM from the HCs group. We also analyzed whether the miRNAs in CSF could discriminate TBM from VM (Supplementary Table 3). The AUC values of the 3 miRNAs (miR-126-3p, miR-130a-3p, and miR-151a-3p) were 0.784 (0.670–0.824), 0.763 (0.646–0.857), and 0.815 (0.703–0.898), respectively, in discriminating TBM from the VM group. miRNAs in CSF does not present better discriminating ability than that in PBMCs.

**Table 3:** ROC analysis of the 4 differentially expressed miRNAs in PBMCs for discriminating TBM from VM groups.

**Signatures**	**AUC (95% CI)**	**Sensitivity (95% CI)**	**Specificity (95% CI)**	**Cutoff value**
**TBM vs. VM**				
hsa-miR-126-3p	0.716 (0.587–0.823)	87.5 (71.0–96.5)	56.7 (37.4–74.5)	
hsa-miR-130a-3p	0.836 (0.721–0.918)	93.7 (79.2–99.2)	70.0 (50.6–85.3)	
hsa-miR-151a-3p	0.762 (0.637–0.861)	96.9 (83.8–99.9)	60.0 (40.6–77.3)	
hsa-miR-199a-5p	0.794 (0.672–0.886)	78.1 (60.0–90.7)	80.0 (61.4–92.3)	
Combination	0.893 (0.788–0.957)	90.6 (75.0–98.0)	86.7 (69.3–96.2)	>0.61^*^
**TBM vs. HCs**				
hsa-miR-126-3p	0.836 (0.723–0.916)	96.8 (83.3–99.9)	61.8 (43.6–77.8)	
hsa-miR-130a-3p	0.827 (0.713–0.910)	93.5 (78.6–99.2)	67.6 (49.5–82.6)	
hsa-miR-151a-3p	0.746 (0.622–0.846)	90.3 (74.2–98.0)	50.0 (32.4–67.6)	
hsa-miR-199a-5p	0.730 (0.605–0.832)	67.7 (48.6–83.3)	70.6 (52.5–84.9)	
Combination	0.878 (0.773–0.946)	93.5 (78.6–99.2)	70.6 (52.5–84.9)	>0.34^*^

*TBM, tuberculous meningitis, n = 32; VM, viral meningitis, n = 30; HCs, healthy controls, n = 34. ROC, receiver operating characteristic curve; AUC, the area under the ROC curve*.

* *The probability value generated in logistic regression with forward stepwise analysis*.

### Independent Validation of the Diagnostic Panel

**Figure 4:**
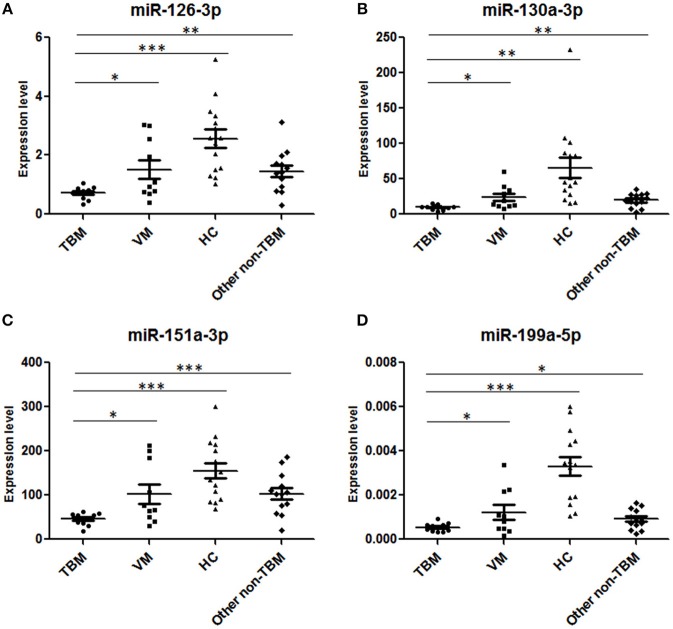
Validation of the 4 miRNAs in the diagnostic panel in the second independent sample set. TBM, tuberculous meningitis, *n* = 11; VM, viral meningitis, *n* = 10; HCs, healthy controls, *n* = 15; non-TBM, *n* = 13. ^*^P < 0.05; ^**^P < 0.01; ^***^P < 0.001; Horizontal bar, the relative expression value of validated miRNAs. The expression values of the validated miRNAs were normalized to the housekeeping miRNA U6 in PBMCs.

**Figure 5:**
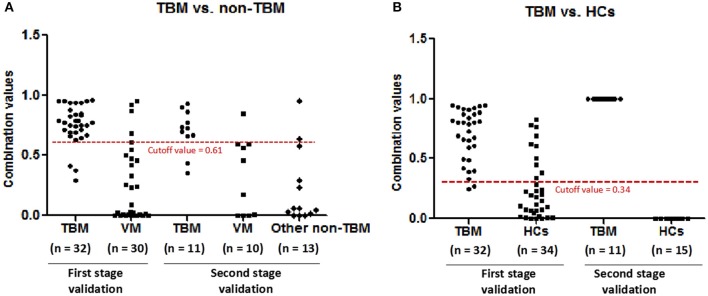
The performance of the diagnostic panel in discriminating TBM patients from VM patients, other non-TBM patients, and HCs. TBM, tuberculous meningitis; VM, viral meningitis; HCs, healthy controls.

In order to validate the diagnostic performance of the 4 miRNA panel, another independent sample set including 11 TBM patients, 10 VM patients, 15 HCs, and 13 other non-TBM patients were further recruited in this study. The expression of the 4 miRNAs in the diagnostic panel was also examined by qPCR. Statistically significant differences of these 4 miRNAs were noted in the TBM group when compared with that in the VM, HCs and other non-TBM group (Supplementary Table 4 and Figures 4A–D). Logistic regression with forward stepwise analysis was also performed to generate the diagnostic panel consisting of the 4 miRNAs. Similar to the first validation set, the diagnostic model effectively discriminated the TBM patients from the other three groups (Supplementary Table 5 and Figure 5). According to the optimal cutoff value yielded in the diagnostic model in the first validation set, it could classify 85.7% individuals (18/21) with a sensitivity of 81.8% (9/11) and a specificity of 90.0% (9/10) in discriminating the TBM patients from the VM patients. Moreover, it showed a sensitivity of 81.8% (9/11), a specificity of 84.6% (11/13), and an accuracy of 83.3% (20/24) in discriminating the TBM group from the other non-TBM group. Furthermore, it presented a sensitivity of 100.0% (11/11), a specificity of 100.0% (15/15), and an accuracy of 100.0% (26/26) when discriminating the TBM group from the HCs group.

## Discussion

TBM is the most serious type of tuberculosis which causes high morbidity and mortality. An accurate and simple diagnosis of TBM is essential to improve TBM treatment and decrease the morbidity and mortality. However, the current available methods for TBM diagnosis presented lower sensitivity, including the culture, conventional ZN, modified ZN with cytospin, and XpertMTB/RIF technology (Heemskerk et al., 2018). Until now, novel biomarker identification is an important approach to increase the sensitivity and specificity of diagnosis. The peripheral blood and cerebrospinal fluid were the routine accessible samples for TBM novel biomarkers screening and validation. Proteins and metabolites have been identified as potential biomarkers for TBM diagnosis (Kumar et al., 2012; Li et al., 2017). Among different molecules, miRNAs were considered more promising candidate signatures based on the better characteristics, including high abundance, stability, ease of sampling and importance as global cellular regulators (Panganiban et al., 2016). In the present study, we conducted a genome-wide miRNA microarray analysis of PBMCs from TBM, VM, and HCs, and further validated in PBMCs and CSF samples in independent validation sets.

Until now, there were only two studies have focused on TBM specific miRNA identification and validation (Pan et al., 2017; Hu et al., 2019), one of which performed microarray assay only between TBM and HCs although they further validated the expression level of the differential miRNAs in non-TBM disease group. Another one study has only validated miR-29 between TBM and HCs in children population, not included non-TBM disease group. The clinicians should discriminate the TBM from other meningitis which is caused by virus or other bacteria, nor the HCs, in clinical practice. Therefore, although the miRNAs was significantly higher or lower in TBM patients than that in HCs, the utility of these miRNAs in clinical practice was limited. The valuable miRNAs should be those that can distinguish TBM and other infectious meningitis. With the declining in incidence of bacterial meningitis due to the use of antibiotics and the excellent tests for cryptococcal meningitis, differentiation between TBM and VM was becoming the most important and urgent issue in recent years, owing to the similar clinical manifestations and difficulties in obtaining etiological evidence. However, no published studies have explored potential miRNAs in distinguishing TBM from VM, and our study was the first study on genome-wide identifying and validating potential miRNA in PBMCs for distinguishing between these two diseases. Four novel miRNAs (miR-126-3p, miR-130a-3p, miR-151a-3p, and miR-199a-5p) were detected between TBM and VM, and three of which were presented difference in both PBMCs and CSF samples. Furthermore, the combination of these 4 miRNAs consistently presented more than 80% accuracy in discriminating the TBM patients from VM patients, HCs and other non-TBM patients, which will be more meaningful in clinical practices.

The expression of miR-126-3p was significantly lower than that in VM and HCs. Previous studies reported that miR-126-3p has a role in protecting blood-brain barrier (BBB) integrity, and inhibit neurons apoptosis and neutrophil infiltration (Xi et al., 2017). It was known that disruption of the BBB is a hallmark of TBM. Therefore, we hypothesized that *M.TB* may down-regulate the expression of miR-126-3p, and further inhibit its protective effect on BBB integrity, and increase neutrophil infiltration to promote inflammation. Previous study has found that the expression level of miR-130a-3p was significantly lower in active TB patients than that in latent TB infection (LTBI) individuals and HCs (Wang et al., 2011), which was consistent with our results that the miR-130a-3p was significantly lower in TBM that that in VM and HCs. However, no study has uncovered the mechanism of miR-130a-3p in *M.TB* infection and further active TB progression. Studies have shown miR-130a/b is expressed in human primary macrophages (Luers et al., 2010), and it was reported that miR-130a could decrease the expression level of TNF-α, and then inhibited the activation of macrophage in *Brucella* infection, indicating the participation of miR-130a in innate immunity after bacteria stimulation (Luo et al., 2018). miR-151a-3p functions as a key regulator in innate immunity, inflammation and epithelial to mesenchymal transition (EMT) (Daugaard et al., 2017; Chen et al., 2018; Liu et al., 2018a), which is consistent with that *M.TB* infection can initially triggers innate immune response and inflammation. LPS stimulation can decrease miR-151-3p expression, and then increase the production of IL-6, indicating that miR-151a-3p may also involve in innate immunity after bacteria stimulation (Liu et al., 2018a). In our study, we also detected that miR-151a-3p was significantly down-expressed in TBM, compared with VM and HCs. Another study has found that miR-151a was a key molecular involved in Th2 cytokine response in atopic dermatitis, and suppressed the expression of Th1 cytokines (Chen et al., 2018). Conversely, Th1 immunity was the main immune response process involved in TBM, so it is likely that the miR-151a was decreased in TBM patients. Recent studies have revealed that pro-inflammatory factors could be regulated by miR-199a, and miR-199a was involved in the development of inflammatory lung diseases (Schickel et al., 2008; Rane et al., 2009; Zuo et al., 2016). It was reported that decreased miR-199a could attenuated the production of pro-inflammatory cytokines in sepsis-induced acute respiratory distress syndrome (Liu et al., 2018b). Pro-inflammatory cytokine production is also critical in active TB, including TBM, so the decreased expression of miR-199a-5p may also play a protective role in TBM progression.

In our study, we detected that miR-130a-3p, miR-424-5p, miR-574-5p, and miR-146a presented significant difference between TBM and HCs in microarray analysis. These 4 miRNAs were also showed significant difference in serum between active TB patients and HCs in previous studies (Fu et al., 2011; Wang et al., 2011, 2016; Meng et al., 2014; Zhou et al., 2016). The overlap of miRNAs between previous studies and our study suggested the reliability of our microarray test results. Until now, only one study has identified differentially expressed miRNAs in plasma exosomes between TBM and HCs (Hu et al., 2019). But the 6 miRNAs identified in the abovementioned study were not detected in our study. This difference may be caused by the different sample types (PBMCs/CSF in our study *vs*. plasma exosomes in previous study) between our study and previous study. Furthermore, the moderate sample size in both our study and previous study may also lead to the different results. However, the validation in both PBMCs and CSF sample sets could confirm the reliability of the differentially expressed miRNAs identified in our study.

In our study, we found that the expression levels of miRNAs in CSF were significantly lower than that in PBMCs, such as miR-199a-5p, which were detected in PBMCs but were not detected in CSF. Although there is no published CSF miRNA profiling study in TBM, the low concentrations of miRNAs in CSF were also detected in other CNS diseases studies, which have found that only 10–30% human miRNAs can be detected in CSF using high-throughput methods or qPCR analysis (Holm et al., 2014; Sorensen et al., 2017). In order to resolve this problem, most of the studies have used pre-amplification step to improve the target abundance without introducing bias, and obtained reliable quantitative detection results (Murray et al., 2016; Lusardi et al., 2017). The reason for the lower miRNAs concentration in CSF may be the presence of the BBB in the nervous system, which is possible to block miRNA entrance to the CSF from the peripheral circulatory system and lead to the lower amount of miRNAs in CSF than that in peripheral blood and human cells. However, due to CSF can directly contact with the CNS, the miRNAs in the CSF may reflect physiology and pathological processes of TBM more accurately than that in other cells or body fluids. Further researches performing high-throughput screening analysis for identifying specific miRNAs for TBM in CSF may better facilitate for TBM diagnosis and deeply understanding of the pathogenesis involved in TBM progression.

Several methods for quantitative detection of CSF miRNA have already been proposed. The commonly used reference molecular are spike-in miRNA (miRNAs originated from *C. elegans* or plant) or endogenous miRNA (Kopkova et al., 2018). The spike-in miRNA should be added to the samples before RNA extraction. Since this exogenous miRNA is normally absent in human cells, tissues and body fluids, its expression levels among samples can reflect the technological variability and can be used as reference controls. The spike-in miRNA control was well-used in several studies involved CSF miRNAs detection (Burgos et al., 2013; McAlexander et al., 2013; Murray et al., 2016). In consistent with previous studies, we detected that the expression level of spike-in miRNA cel-miR-39-3p were stable among all the samples and can be used as the reference molecular in our study. Previous studies also used stable endogenous miRNAs or another short RNA. Recent works have also identified several endogenous controls in CSF, which is not associated with the pathogenic condition. For instance, U6 snRNA, RNU44, miR-16, miR-24, miR-21, and let-7c, et al. (Baraniskin et al., 2011; Teplyuk et al., 2012; Muller et al., 2014; Marques et al., 2017; Pan et al., 2017) were used in other CNS diseases. In our study, we also analyzed the expression levels of U6 snRNA in samples based on the fixed RNA reverse transcription amount. However, the results showed that the U6 snRNA was not stable and not suitable for CSF miRNA normalization in qPCR analysis in tuberculous and viral meningitis. The pathological mechanisms and processes of neurological diseases vary greatly, which may be one of the important reasons leading to the difference in expression levels of endogenous miRNAs in CSF in different CNS diseases. Therefore, it is very important to choose the best reference molecular in miRNA quantitative detection in CSF, and the traditional reference miRNAs used in human cells, tissues or other body fluids may not be stable molecule in CSF miRNA quantitative detection.

To our knowledge, this is the first study to identify candidate miRNAs for distinguishing TBM from VM using high-throughput microarray analysis and further validated in independent PBMCs and CSF samples. However, there are still some limitations in our study. First, since we focused on distinguishing between TBM and VM due to the lack of etiological evidence in these two diseases, other disease controls were not recruited in the microarray stage, although we have recruited 13 other non-TBM patients in the second validation stage. Since only a few cases of non-TBM have been validated in our study, and not all the categories of central neural system diseases were included, we cannot rule out that maybe some other diseases in central neural system can also deregulate these miRNAs. Therefore, further studies including larger cases are needed to determine the specificity of these 4 miRNAs panel in the diagnosis of TBM. Furthermore, since the incidence of TBM is lower and the definite diagnosis of TBM faces huge difficulties in our country, the number of samples in our study was not large enough, especially in the microarray test. We cannot rule out the effect of inter-individual differences in the microarray set. As shown in Figure 1, the expression profile of one sample in VM group and one sample in HCs group was looked like different from other three samples in the same group, although the clustering results were coincident with the disease status. This difference may be caused by the heterogeneity among samples in each group. Although additional analysis showed that most of the differentially expressed miRNAs were overlapped between the analysis with or without these 2 samples, and the expression level of the 4 miRNAs in the diagnostic panel were consistently presented significant difference between TBM and other groups, we cannot rule out that much more samples in microarray test will reduce the heterogeneity effect. In addition, although two independent sample sets were recruited to validate the diagnostic performance of the differentially expressed miRNA, the sample size in each independent set was not large enough. Future study including larger sample sets should be conducted to validate our results.

In conclusion, our study uncovered the miRNA profiles of TBM and VM patients, and firstly identified 4 differentially expressed miRNAs between TBM and VM. Furthermore, a new diagnostic model consisting of these 4 miRNAs was constructed and displayed a relatively better ability in distinguishing TBM from VM. These results not only presented a new potential diagnostic signature in discriminating TBM and VM, but also helpful to better understand the pathogenesis involved in the TBM.

## Data Availability

The datasets generated for this study can be found in the Gene Expression Omnibus, GSE131708.

## Ethics Statement

The studies involving human participants were reviewed and approved by the Ethics Committee of the Beijing Chest Hospital, Capital Medical University. The patients/participants provided their written informed consent to participate in this study.

## Author Contributions

LP, FL, and ZZ designed the experiments. LP, HJ, RW, BD, QS, and AX conducted the experiments. FL, JZ, JL, MH, XL, WC, ZD, and YW enrolled the subjects. LP analyzed the data and wrote the paper.

### Conflict of Interest Statement

The authors declare that the research was conducted in the absence of any commercial or financial relationships that could be construed as a potential conflict of interest.
