# Supplementary material for: Genome-Wide miRNA Analysis Identifies Potential Biomarkers in Distinguishing Tuberculous and Viral Meningitis
Source: Front Cell Infect Microbiol. 2019 Sep 10;9:323. doi: 10.3389/fcimb.2019.00323 (PMC6749153; doi:10.3389/fcimb.2019.00323)
Supplement: Supplementary file 1 [file Data_Sheet_1.docx]

**Supplementary Table 1. Differentially expressed miRNA in the comparison of TBM with VM and HCs groups.**

| **No.** | **miRNAs** | **TBM vs. VM** | | **TBM vs. HCs** | |
| --- | --- | --- | --- | --- | --- |
|  |  | **Fold change** | ***P*-value** | **Fold change** | ***P*-value** |
| **1** | **hsa-miR-126-3p** | **0.220** | **0.003** | **0.457** | **0.014** |
| **2** | **hsa-miR-130a-3p** | **0.244** | **0.006** | **0.518** | **0.041** |
| **3** | **hsa-miR-151a-3p** | **0.418** | **0.033** | **0.589** | **0.047** |
| **4** | **hsa-miR-199a-5p** | **0.386** | **0.015** | **0.624** | **0.042** |
| **5** | **hsa-miR-642a-3p** | **1.656** | **0.018** | **1.842** | **0.008** |
| **6** | **hsa-miR-4299** | **1.611** | **0.026** | **1.554** | **0.014** |
| 7 | hsa-let-7e-5p | 0.454 | 0.023 | 0.711 | 0.236 |
| 8 | hsa-miR-103a-3p | 0.644 | 0.009 | 0.935 | 0.620 |
| 9 | hsa-miR-1273g-3p | 1.580 | 0.001 | 1.463 | 0.003 |
| 10 | hsa-miR-146b-5p | 1.923 | 0.003 | 1.164 | 0.212 |
| 11 | hsa-miR-150-5p | 1.670 | 0.003 | 0.970 | 0.643 |
| 12 | hsa-miR-151a-5p | 0.425 | 0.030 | 0.757 | 0.332 |
| 13 | hsa-miR-15b-5p | 0.626 | 0.005 | 1.197 | 0.401 |
| 14 | hsa-miR-185-5p | 0.534 | 0.001 | 0.740 | 0.024 |
| 15 | hsa-miR-197-5p | 1.656 | 0.009 | 1.429 | 0.046 |
| 16 | hsa-miR-199a-3p | 0.266 | 0.004 | 0.638 | 0.101 |
| 17 | hsa-miR-21-5p | 0.612 | 0.007 | 1.153 | 0.328 |
| 18 | hsa-miR-221-3p | 0.563 | 0.036 | 0.891 | 0.618 |
| 19 | hsa-miR-223-3p | 0.641 | 0.034 | 1.474 | 0.032 |
| 20 | hsa-miR-23b-3p | 0.520 | 0.019 | 0.803 | 0.230 |
| 21 | hsa-miR-24-3p | 0.636 | 0.011 | 0.831 | 0.210 |
| 22 | hsa-miR-27b-3p | 0.465 | 0.013 | 0.786 | 0.216 |
| 23 | hsa-miR-324-5p | 0.660 | 0.028 | 0.912 | 0.459 |
| 24 | hsa-miR-3653 | 1.769 | 0.017 | 0.938 | 0.703 |
| 25 | hsa-miR-3960 | 1.613 | 0.032 | 0.667 | 0.365 |
| 26 | hsa-miR-6087 | 1.570 | 0.038 | 1.316 | 0.008 |
| 27 | hsa-miR-6125 | 1.505 | 0.033 | 1.073 | 0.541 |
| 28 | hsa-miR-6127 | 1.998 | 0.001 | 1.416 | 0.114 |
| 29 | hsa-miR-324-3p | 1.459 | 0.071 | 1.502 | 0.034 |
| 30 | hsa-miR-424-5p | 1.109 | 0.589 | 1.655 | 0.028 |
| 31 | hsa-miR-451a | 3.825 | 0.106 | 6.064 | 0.033 |
| 32 | hsa-miR-574-5p | 0.839 | 0.232 | 0.523 | 0.027 |

TBM, tuberculous meningitis, n = 4; VM, viral meningitis, n = 4; HCs, healthy controls, n = 4.

The 6 overlapping miRNAs in the comparison of TBM with VM and HCs were in bold.

**Supplementary Table 2. Validation of the differential miRNAs in the first independent sample set.**

| **miRNA** | **Validation in PBMCs*** |  | |  |  | | **Validation in CSF^#^** |  |
| --- | --- | --- | --- | --- | --- | --- | --- | --- |
|  | **Fold Change (TBM/VM)** | ***P*-value** | **Fold Change (TBM/HCs)** | | | ***P*-value** | **Fold Change (TBM/VM)** | ***P*-value** |
| hsa-miR-126-3p | 0.466 | 0.00054 | 0.432 | | | 3.24E-06 | 0.336 | 0.017 |
| hsa-miR-130a-3p | 0.360 | 7.28E-06 | 0.511 | | | 2.05E-05 | 0.216 | 0.0042 |
| hsa-miR-151a-3p | 0.427 | 0.00015 | 0.556 | | | 0.00023 | 0.303 | 0.00074 |
| hsa-miR-199a-5p | 0.357 | 3.75E-05 | 0.402 | | | 0.0035 | NA | NA |
| hsa-miR-642a-3p | 1.059 | 0.758 | 1.256 | | | 0.261 | - | - |
| hsa-miR-4299 | 1.208 | 0.223 | 1.923 | | | 0.00075 | **-** | **-** |

TBM, tuberculous meningitis; VM, viral meningitis; HCs, healthy controls.

* Validation in PBMCs was performed in 32 TBM, 30 VM and 34 HCs.

**^#^** Validation in CSF was performed in 36 TBM and 34VM patients.

**Supplementary Table 3. ROC analysis of the 3 differentially expressed miRNAs in CSF for discriminating TBM from VM groups.**

| **Signatures** | **AUC (95% CI)** | **Sensitivity (95% CI)** | **Specificity (95% CI)** |
| --- | --- | --- | --- |
| hsa-miR-126-3p | 0.784 (0.670 – 0.874) | 72.2 (54.8 – 85.8) | 64.7 (46.5– 80.3) |
| hsa-miR-130a-3p | 0.763 (0.646 – 0.857) | 91.7 (77.5 – 98.2) | 50.0 (32.4 – 67.6) |
| hsa-miR-151a-3p | 0.815 (0.703 – 0.898) | 83.3 (67.2 – 93.6) | 69.7 (51.3 – 84.4) |

TBM, tuberculous meningitis, n = 36; VM, viral meningitis, n = 34.

ROC, receiver operating characteristic curve; AUC, the area under the ROC curve.

**Supplementary Table 4. Validation of the 4 miRNAs in diagnostic panel in the second independent sample set.**

| **miRNAs** | **TBM vs. VM** | | **TBM vs. HCs** | | **TBM vs. other non-TBM** | |
| --- | --- | --- | --- | --- | --- | --- |
|  | **Fold change** | ***P*-value** | **Fold change** | ***P*-value** | **Fold change** | ***P*-value** |
| hsa-miR-126-3p | 0.471 | 0.019 | 0.279 | 3.15E-05 | 0.491 | 0.0032 |
| hsa-miR-130a-3p | 0.408 | 0.013 | 0.146 | 0.0028 | 0.490 | 0.0033 |
| hsa-miR-151a-3p | 0.444 | 0.014 | 0.296 | 1.99E-05 | 0.447 | 0.0009 |
| hsa-miR-199a-5p | 0.445 | 0.047 | 0.164 | 7.18E-06 | 0.595 | 0.019 |

TBM, tuberculous meningitis, n = 11; VM, viral meningitis, n = 10; HCs, healthy controls, n = 15; Non-TBM, n = 13.

Validation of the 4 miRNAs in PBMCs.

**Supplementary Table 5. ROC analysis of the diagnostic panel consisting of 4 miRNAs in discriminating TBM from VM, HCs and other non-TBM in the second independent sample set.**

|  | **AUC (95% CI)** | **Sensitivity (95% CI)** | **Specificity (95% CI)** |
| --- | --- | --- | --- |
| TBM vs. VM | 0.855 (0.633 – 0.968) | 81.8 (48.2 – 97.7) | 90.0 (55.5 – 99.7) |
| TBM vs. HCs | 1.000 (0.868 – 1.000) | 100.0 (71.5 – 100.0) | 100.0 (78.2 – 100.0) |
| TBM vs. other non-TBM | 0.902 (0.711 – 0.985) | 81.8 (48.2 – 97.7) | 84.6 (54.6 – 98.1) |

TBM, tuberculous meningitis, n = 11; VM, viral meningitis, n = 10; HCs, healthy controls, n = 15; Non-TBM, n = 13.

ROC, receiver operating characteristic curve; AUC, the area under the ROC curve.

The diagnostic panel was generated by logistic regression with forward stepwise analysis in the basis of the expression values of the 4 miRNAs.

**Supplementary Figures**


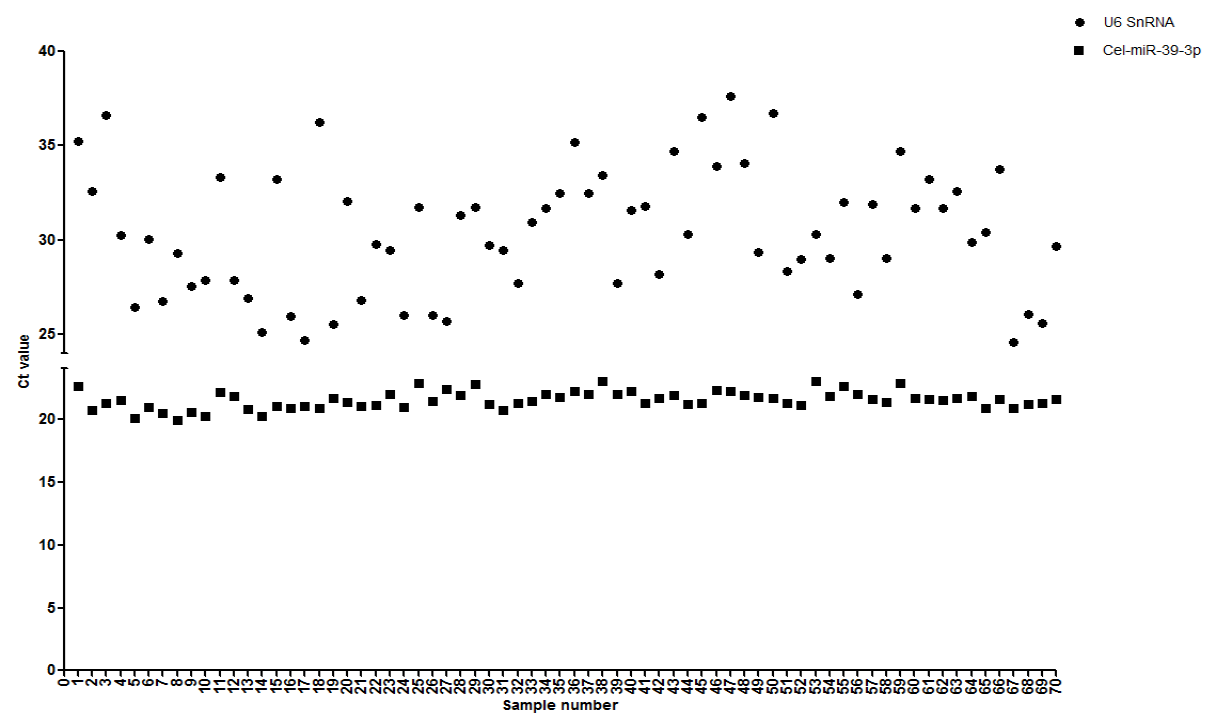


**Supplementary Figure 1. The expression levels of U6 snRNA and cel-miR-39-3p in 70 CSF samples.**

Horizontal bar, the cycle threshold (Ct) value in qPCR analysis.
